# A scoping review of the perceptions of death in the context of organ donation and transplantation

**DOI:** 10.1186/s12910-021-00734-z

**Published:** 2021-12-18

**Authors:** George Skowronski, Anil Ramnani, Dianne Walton-Sonda, Cynthia Forlini, Michael J. O’Leary, Lisa O’Reilly, Linda Sheahan, Cameron Stewart, Ian Kerridge

**Affiliations:** 1 Sydney Health Ethics, University of Sydney, Sydney, Australia; 2 Canberra Hospital, Canberra, Australia; 3 ACT Health Library, Canberra, Australia; 4 School of Medicine, Deakin University, Geelong, Australia; 5 Royal Prince Alfred Hospital, Sydney, Australia; 6 South East Sydney Local Health District, Sydney, Australia; 7 Law School, University of Sydney, Sydney, Australia; 8 Haematology Department, Royal North Shore Hospital, St Leonards, Australia; 9 St George Hospital, Gray Street, Kogarah, NSW 2217 Australia

## Abstract

**Background:**

Socio-cultural perceptions surrounding death have profoundly changed since the 1950s with development of modern intensive care and progress in solid organ transplantation. Despite broad support for organ transplantation, many fundamental concepts and practices including brain death, organ donation after circulatory death, and some antemortem interventions to prepare for transplantation continue to be challenged. Attitudes toward the ethical issues surrounding death and organ donation may influence support for and participation in organ donation but differences between and among diverse populations have not been studied.

**Objectives:**

In order to clarify attitudes toward brain death, organ donation after circulatory death and antemortem interventions in the context of organ donation, we conducted a scoping review of international English-language quantitative surveys in various populations.

**Study appraisal:**

A search of literature up to October 2020 was performed, using multiple databases. After screening, 45 studies were found to meet pre-specified inclusion criteria.

**Results:**

32 studies examined attitudes to brain death, predominantly in healthcare professionals. In most, around 75% of respondents accepted brain death as equivalent to death of the person. Less common perspectives included equating death with irreversible coma and willingness to undertake organ donation even if it *caused* death. 14 studies examined attitudes to organ donation following circulatory death. Around half of respondents in most studies accepted that death could be confidently diagnosed after only 5 min of cardiorespiratory arrest. The predominant reason was lack of confidence in doctors or diagnostic procedures. Only 6 studies examined attitudes towards antemortem interventions in prospective organ donors. Most respondents supported minimally invasive procedures and only where specific consent was obtained.

**Conclusions:**

Our review suggests a considerable proportion of people, including healthcare professionals, have doubts about the medical and ethical validity of modern determinations of death. The prognosis of brain injury was a more common concern in the context of organ donation decision-making than certainty of death.

**Supplementary Information:**

The online version contains supplementary material available at 10.1186/s12910-021-00734-z.

## Background

Longstanding and almost universal socio-cultural perceptions surrounding death were radically changed by the more-or-less simultaneous emergence of several medical technologies in the 1950s–60s. These included mechanical ventilation and the development of modern intensive care units, cardiopulmonary resuscitation and defibrillation.

The concept of brain death (BD) has not been accepted without controversy [1]. Concerns revolve around the fundamental question of whether brain death is a manifestation of biological death, but also in regard to the clinical process by which brain death is determined and whether there is a requirement to confirm death of the whole brain. Over the decades there have been numerous publications on these matters [2, 3]. While most of the debate has been conducted in the scientific and ethical literature, in recent years there have been a number of international legal challenges to its validity in individual cases [4, 5]. Documentation of the World Brain Death Project [6], developed by international consensus, has improved uniformity in the diagnostic process for BD, but it has done little to address fundamental philosophical questions around its meaning and significance [7].

Although early transplants involved donors whose heartbeat and breathing had ceased, brain death subsequently became the predominant path to organ donation, as it increased both the range and quality of donated organs. However, since around 2005 there has been a resurgence of interest in utilising donors dying following cardio-respiratory failure because (1) the rapidly increasing demand for transplantation greatly exceeded the supply of suitable organs from BD donors, and (2) it was anticipated that peri-mortem retrieval of vital organs for transplantation from people declared dead following circulatory failure would not violate what has become known as the ‘dead donor rule’ (DDR)—the notion that vital organs can only be removed from persons who have *already* been declared dead [8]. ‘Donation after Circulatory Determination of Death’ (DCDD) requires that organ retrieval occurs rapidly, before irreversible ischaemic injury can supervene, but in order to confirm that death has occurred prior to commencement of retrieval surgery, strict time constraints around the cardio-respiratory signs of death are imposed, based on the likelihood of auto-resuscitation.

To mitigate against ischaemic damage and improve outcomes following organ transplantation a range of interventions with varying degrees of invasiveness, which are not part of usual end-of-life care, can be undertaken in DCDD patients prior to the declaration of death. While these so-called “antemortem interventions” are permitted in some jurisdictions, relying ethically on arguments linking them with the patient’s best interests based on their “interest” in being a donor, they are controversial because they are arguably more frequently performed primarily in the organ recipient’s interests, rather than those of the donor in the context of quality end of life care.

Studies that have examined attitudes to BD have generally been small and confined to restricted professional, cultural and regional populations. Many studies also tend to conflate support for OD and physiological comprehension of BD with moral ‘support’ for BD and DCDD, assuming that any concerns about them reflect a knowledge gap rather than a values-based rejection [9–14]. Consequently, individual studies may fail to provide unbiased and comprehensive accounts of the range of ethical views regarding BD or DCDD and related attitudes in relevant communities. Both of these perspectives are needed to ensure that policies and protocols around brain death, DCDD and organ donation are consistent with the values and attitudes of donors, healthcare professionals and the general public. In order to clarify these perspectives, we conducted a scoping review of studies that have examined the acceptance and understanding of BD and DCDD, including related antemortem interventions, in various populations, and their relation to decision-making in the context of organ donation.

## Methods

### Research methodology

A scoping review methodology was chosen because it accommodates the heterogeneity in study aims and methods used in international studies examining the acceptance and understanding of BD and DCDD in the context of organ transplantation. Additionally, while systematic reviews require methodological uniformity and are most useful where outcomes measures are easily defined and measured, scoping reviews can reveal areas of divergence and debate, identify gaps in what is known about a field, issue or question and enable exploration of underlying or foundational concepts or ideas [15, 16]. The scoping review was guided by the PRISMA protocol with conceptualisation of the research question as ”What quantitative evidence is available regarding the acceptance of and attitudes towards the concepts of BD, DCDD and the DDR, and how these relate to attitudes and decision-making regarding organ donation?" Assessment of relevance was done following the ‘methodology-issue-participant approach’ described by Strech et al. [17] (Table 1).

**Table 1:** Inclusion and exclusion criteria applied to studies in the ‘eligibility’ step of the PRISMA protocol

	Inclusion criteria	Exclusion criteria
Study characteristics	Written in English Published as full-text article in an indexed journal Sufficient details about methodology and results available	Discussion or review articles Studies using qualitative methodology Articles published in a language other than English
Participants	Members of the general public Students regardless of discipline Healthcare professionals	
Data	Studies reporting *quantitative* data on the attitudes and beliefs of relevant populations on brain death, circulatory death, and perimortem interventions, in the context of organ donation	Studies Only testing *knowledge* or *awareness* of concepts related to brain death, circulatory death, dead donor rule, organ donation Studies reporting data on emotional responses to death as an event Studies collecting data on attitudes toward organ donation alone

### Literature search

The search strategy included a combination of synonyms and controlled vocabularies from Medical Subject Headings (MeSH), EmTree, Thesaurus of Psychological Index Terms and CINAHL Subject Headings. The search was conducted on Medline (OVID) and replicated using Embase (OVID), PubMed, EmCare for Nursing (OVID), PsycINFO (OVID), Cochrane and CINAHL databases using truncations and Boolean operators.

The full search strategy is detailed in the Additional file 1.

Studies were identified by database searches following deduplication. The studies were screened by the authors on the basis of their abstracts, which were then filtered for relevance according to predetermined inclusion and exclusion criteria by two authors per article (Table 1). Disagreements were reconciled by discussion within pairs or by a third author if disagreements persisted.

Hand searches of the grey literature and of reference lists in relevant articles were also performed in order to minimise the risk of missed studies.

### Date limits

The study examined papers published up to October 2020, with the earliest appearing in 1972.

### Quality assessment

Methodologic quality was assessed using a checklist proposed by Roever [18]. Each study was assessed independently by two authors. Studies meeting the inclusion criteria were further subjected to risk of bias assessment based on criteria developed by Agarwal et al. [19] Because of the heterogeneity of study populations, methods, instruments and outcome measures, formal meta-analysis was not conducted.

## Results

**Fig. 1:**
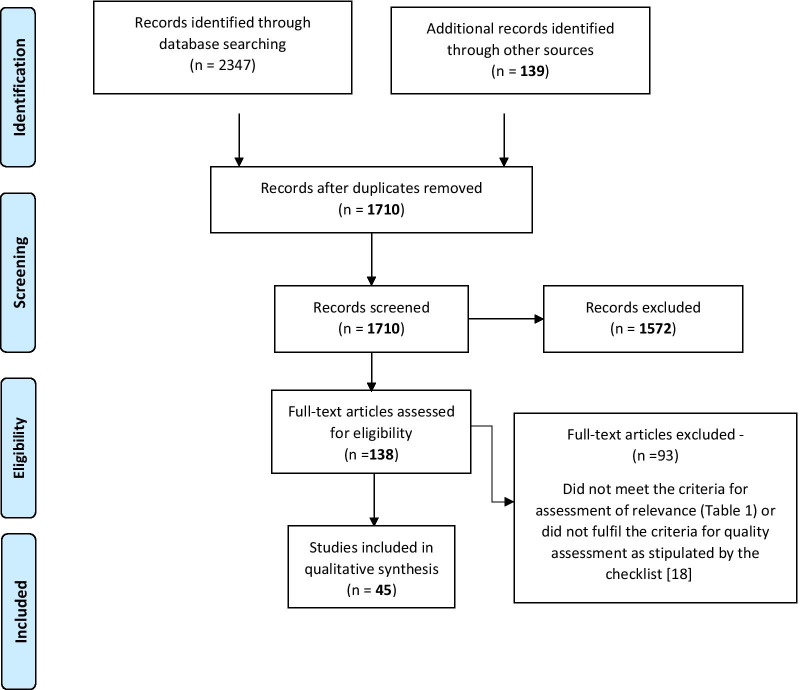
PRISMA flow chart

An initial database search yielded 2347 abstracts. A further 139 were identified from other sources. After screening, 138 papers were reviewed in detail (Fig. 1). This yielded a final list of 45 included studies.

**Fig. 2:**
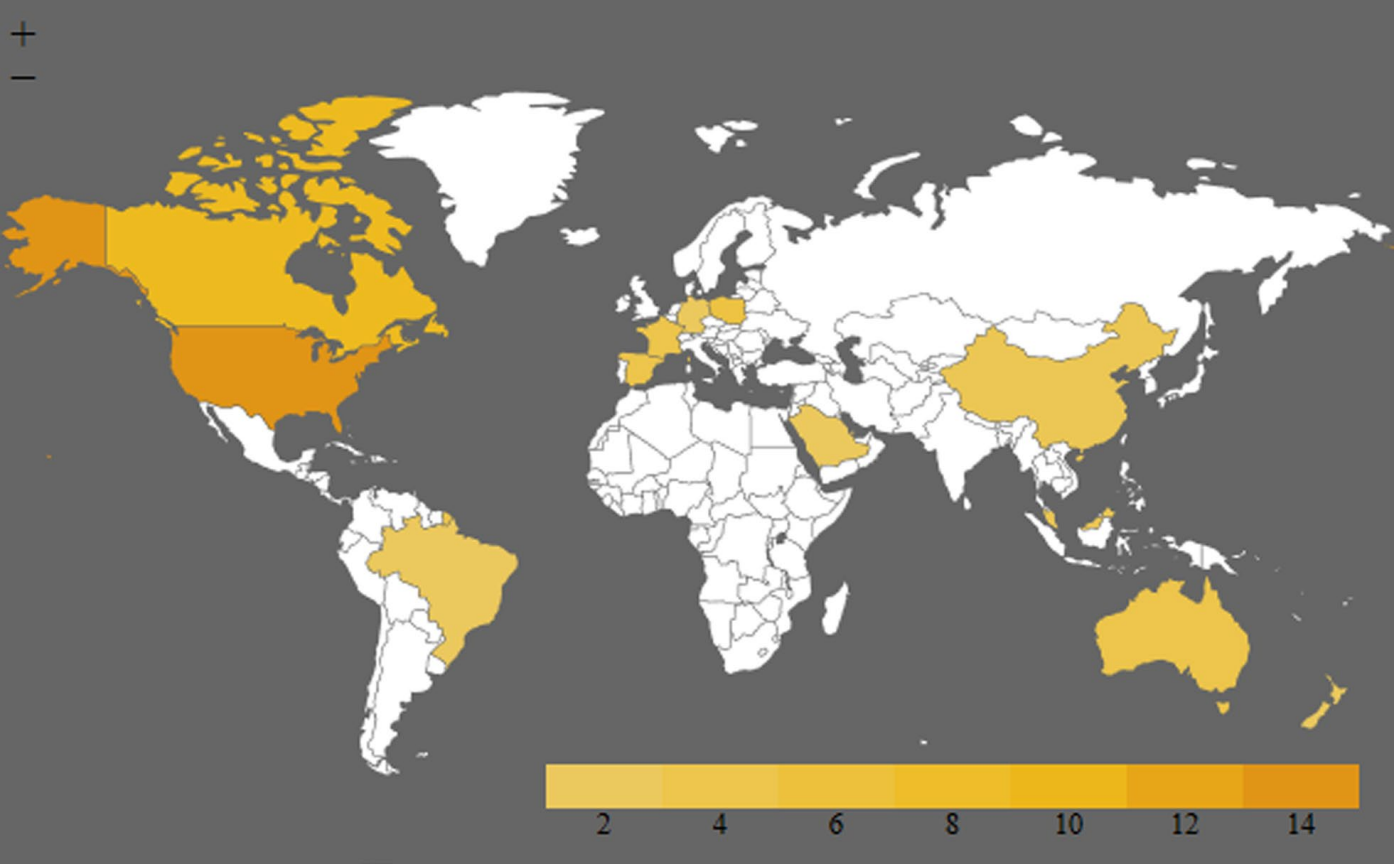
Geographical distribution of studies identified

Table 2 shows the risk of bias assessment for the 45 included studies. 34 studies were assessed as having a low or very low risk of bias whereas 12 studies had a high risk of bias. This assessment indicates that the empirical evidence included in our scoping review is overall of good quality. However, there is a strong preponderance of studies from North America (Fig. 2).

**Table 2:** Risk of bias assessment

Study year location	Sample size	Representativeness of the sample Y/N or unclear. (Y if randomisation or stratified or systematic sampling technique was used or majority of population in question was targeted.)	Adequacy of response rate: excellent, good, average, poor or data not shared (> 75, > 50, > 25, > 0%)	Missing data	Conduct of pilot testing: Y/N (If no mention considered not done)	Established validity of survey instruments: Y/N (If no mention considered not done)	Overall risk of bias
Alsaied 2012 Qatar [10]	418	Y	Good	Combined with non-responders so unclear (total 28.5%)	Y	Unclear	Low risk
Burroughs 1998 USA [13]	225	Y	Excellent	No loss	Unclear (mentions questionnaire was refined)	Unclear (mentions questionnaire was refined)	Low risk
Camut 2016 France [50]	174	Unclear how the participants were targeted	Good	No loss	Y	N	High risk
Cohen 2008 Israel [25]	2366	Y	Good	Minimal overall less than 10%	Y^a^	Y	Very low risk
DeJong 2013 Canada [43]	189	N (administered at a public festival and $5 incentive)	No data	Minimal	Y	Y	High risk
Dhanani et al. 2012 Canada [40]	245	Y	Average	Minimal	Y	Y	Very low risk
Dubois et al. 1999 USA [23]	613	Y	Average	Minimal	Y	Unclear	Low risk
ElSafi et al. 2017 Saudi Arabia [26]	434	Y (single centre but good numbers)	Excellent	Minimal	Y	Y	Very low risk
Floden 2011 Sweden [9]	702	Y	Good	Minimal	N	Partial validity	Low risk
Goudet 2013 France [44]	1057	Y	Average	11.60%	N	N	Low risk
Hart et al. 2012 USA [45]	1122	Y	Average but non-response bias studied and excluded	Minimal as questionnaires with more than 80% of response were included	Y	Y	Very low risk
Health professionals survey Canada 2006 [51]	720	N	Poor	< 15%^b^	N	Y	High risk
Honarmand 2020 Canada [59]	398	Non- randomized (self-selection bias)	21.2	Incomplete surveys excluded	Y	Y	Low risk
Hu 2015 China [51]	373	Adopted randomisation	Excellent	None	Y	Y	Very low risk
Hyde et al. 2011 Australia [31]	468	N (possible snowballing of email, students enrolled in a particular subject were targeted, most likely for convenience)	Poor for public and average for uni students	Minimal	N	N	Very high risk
Iriarte 2012 Spain [32]	828	Unclear (single university and demographics not shared)	Not shared	Not shared but apparently minimal	N	N	High risk
Joffe et al. 2008 Canada [41]	80	N (single centre)	Good	Minimal	Y	Y	Very low risk
Joffe et al. 2008 Canada [46]	318	N (medical Ethics and philosophy students only)	Excellent	Minimal	Y	Y	Very low risk
Joffe et al. 2012 USA [22]	192	Y	Average	12% (were excluded from the analysis)	Y	Maybe	Very low risk
Keenan et al. 2002 Canada [56]	128	Y for public but not for healthcare workers	Not shared	Apparently minimal	Y	Y	Low risk
Kubler et al. 2009 Poland [33]	1128	Y	Unclear	Minimal if any	N	N (translated but not validated in Polish)	Low risk
Lee et al. 2018 Australia [57]	161	Non randomized (self selection bias)	Between 24 and 37%	Responses with missing data excluded	N	Y (content validity by expert panel)	Low risk
Lewis et al. 2020 USA [60]	92	Non- randomized (self-selection bias)	92/2460	Appears minimal	N	N	High risk
Lomero et al. 2015 Spain [24]	236	Single centre	Good	Minimal	Y	Y	Very low risk
Mathur et al. 2008 USA [42]	157	Single centre	Excellent/good (pre and post)	< 10%	N	Y	Low risk
Marck et al. 2012 Australia [30]	811	Y	Poor	Minimal	N	Y	High risk
Marcum 2002 USA [14]	229	Y	Excellent	Minimal	N	Y	Very low risk
Mikla et al. 2015 Poland [11]	492	Y (single university but attempts made to select from all levels of training)	Excellent	Minimal 93% completion rate	Y	Y	Very low risk
Nair-Collins et al. 2015 USA [49]	1096	Y	Excellent	Minimal	Y	Y	Very low risk
Nasrollahzadeh et al. 2003 Iran [27]	130	N (130 nurses from 10 ICUs is a small proportion without randomization)	Excellent	Minimal	Y	Y	Low risk
Nowak et al. 2014 Poland [34]	800	Unclear (stratification medical vs non-medical, but non-medical demographics skewed towards female sex by a ratio of 3:1)	Seems 100% but unclear	Minimal	N	N	High risk
Oo et al. 2020’ Malaysia [61]	412	HCW working in ED ICU and Neuro Sx	98%	6%	N	Y	Low risk
Othman et al. 2020 International [38]	1072	Public (self selection bias)	–	Minimal	N	N	High risk
Public survey Canada 2005 [37]	1505	Unclear	Not shared	Unclear	N	N	High risk
Rodrigue et al. 2018 USA [48]	112	N (single transplant centre)	Good	Minimal	Y	Y	Low risk
Rodriguez-Arias 2013 Spain France USA [47]	587	Y	Average	Minimal	Y	Y	Very low risk
Roels et al. 2010 Multiple countries [20]	19,537	Yes	Good	Not mentioned	Y	Y	Very low risk
Rozaidi et al. 2000 Malaysia [28]	426	Unclear	Unclear	Minimal	N	N	Very high risk
Sarnaik et al. 2013 USA [39]	264	N (73.4% working in a transplant centre suggesting response bias)	Average	Minimal	Y	N	Low risk
Schicktanz et al. 2017 Germany [35]	648	Unclear (some attempt at stratification)	Good	Minimal	N	Maybe (comprehensibility tested)	Low risk
Siminoff et al. 2004 USA [36]	1351	Y	Good	Minimal	Y	Y	Very low risk
Skwirczyńska et al. 2019 Poland [58]	368	Non- randomized (self-selection bias)	73.6	Unstated	Y	Y (previously validated and extensively used)	Low risk
Teixeira et al. 2012 Brazil [12]	136	Single centre	Unclear	Minimal	N	N	High risk
Yang et al. 2015 China [29]	476	N (convenience sampling)	Excellent	Some	N	Y	Low risk
Youngner et al. 1989 USA [21]	195	Unclear (one group was randomized not the other)	Excellent	Minimal	Y	Y	Very low risk

https://www.evidencepartners.com/wp-content/uploads/2017/04/Methods-Commentary-Risk-of-Bias-in-cross-sectional-surveys-of-attitude....pdf

^a^previously extensively used questionnaire by the Eurotransplant Organización International

^b^Pertaining to the sections reviewed for this study

Table 3 summarises the themes explored by included studies, and Table 4 lists the main findings of each.

**Table 3:** Included studies and overview of themes explored

Author/year	n	Target population studied	Country	Belief in brain death criteria	Belief in DCDD criteria	Dead donor rule	Ante mortem interventions/consent
Alsaied 2012 [10]	418	HCW	Qatar	✓			
Burroughs 1998 [13]	225	Public	USA	✓			
Camut 2016 [50]	174	HCW	France	✓			✓
Cohen 2008 [25]	2366	HCW	Israel	✓			
DeJong 2013 [43]	189	Public	Canada		✓	✓	
Dhanani et al. 2012 [40]	245	HCW	Canada		✓		✓
Dubois et al. 1999 [23]	613	HCW	USA	✓	✓		
ElSafi et al. 2017 [26]	434	Students	Saudi Arabia	✓			
Floden 2011 [9]	702	HCW	Sweden	✓			
Goudet 2013 [44]	1057	HCW	France		✓		✓
Hart et al. 2012 [45]	1122	HCW	USA		✓		
Health professionals survey 2006 [51]	720	HCW	Canada		✓		✓
Honarmand et al. [59]	398	HCW	Canada				✓
Hu 2015 [55]	373	HCW	China	✓			
Hyde et al. 2011 [31]	468	Public & Students	Australia	✓			
Iriarte 2012 [32]	828	Students	Spain	✓			
Joffe et al. 2008 [22]	80	HCW	Canada		✓		
Joffe et al. 2008 [46]	318	Students	Canada		✓		
Joffe et al. 2012 [41]	192	HCW	USA	✓			
Keenan et al. 2002 [56]	128	HCW and Public	Canada		✓		
Kubler et al. 2009 [33]	1128	HCW & Students	Poland	✓		✓	
Lee et al. [57]	161	HCW	AUS-NZ				✓
Lewis et al. 2020 [60]	92	HCW	USA	✓			
Lomero et al. 2015 [24]	236	HCW	Spain	✓			
Mathur et al. 2008 [42]	157	HCW	USA		✓		
Marck et al. 2012 [30]	811	HCW	Australia	✓			
Marcum 2002 [14]	229	HCW	USA	✓			
Mikla et al. 2015 [11]	492	Students	Poland	✓			
Nair-Collins et al. 2015 [49]	1096	Public	USA	✓		✓	
Nasrollahzadeh et al. 2003 [27]	130	HCW	Iran	✓			
Nowak et al. 2014 [34]	800	Students	Poland	✓			
Oo et al. 2020 [61]	412	HCW	Malaysia	✓			
Othman et al. [38]	1072	Public	Europe and North America	✓	✓		
Public survey 2005 [37]	1505	Public	Canada		✓		✓
Rodrigue et al. 2018 [48]	112	HCW	USA		✓		
Rodriguez-Arias 2013 [47]	587	HCW	Spain France USA	✓			
Roels et al. 2010 [20]	19,537	HCW	multiple countries	✓			
Rozaidi et al. 2000 [28]	426	HCW	Malaysia	✓			
Sarnaik et al. 2013 [39]	264	HCW	USA	✓		✓	✓
Schicktanz et al. 2017 [35]	648	Students	Germany	✓		✓	
Siminoff et al. 2004 [36]	1351	Public	USA	✓		✓	
Skwirczyńska et al. 2019 [58]	368	HCW	Poland	✓	✓		
Teixeira et al. 2012 [12]	136	Public	Brazil	✓			
Yang et al. 2015 [29]	476	HCW & Students	China	✓			
Youngner et al. 1989 [21]	195	HCW	USA	✓

**Table 4:** Included studies—main findings

Author + location	Aim	Findings
Alsaied Qatar 2012 [10]	To identify and assess the level of knowledge and attitudes of health care professionals (HCP) in Qatar toward organ donation and transplantation	46.8% physicians believe BD equivalent to death, the figure for nurses was 18.2% and that for EMS technicians was 47.5%. Less than half the subjects were aware that brain death was legal in Qatar
Burroughs USA 1998 [13]	To examine the psychological consequences of consenting or refusing donation of the organs or tissue of a dying family member	Families who were satisfied with their decision to donate were more likely to have understood brain death or had it explained to them as compared to non-donors or non-satisfied donors. Individuals who felt pressured were less likely to donate
Camut France 2016 [50]	To investigate the feelings and the acceptance in healthcare professionals of non-therapeutic intensive care for brain death organ donation and to assess their training needs	8.3% of HCW do not regard brain death as true death. Overwhelming majority support non-therapeutic Intensive Care in the context of organ preservation for donation. However, > 75% favour advance patient's consent and approval of family
Cohen Israel 2008 [25]	Whether attitude to brain death of health care professionals influences the organ retrieval process	78.9% had a positive attitude towards brain death which translated into more comfort with various practical aspects of donation process
DeJong Canada 2013 [43]	To determine public opinion regarding whether DCDD donors are dead at the time of organ retrieval	68% of respondents believed death had occurred after 5 min of absent circulation with prior DNR in place. In the absence of DNR that figure dropped to 53%. 49% said dead donor rule should be discarded
Dhanani et Canada 2012 [40]	To describe the manner in which Canadian adult and paediatric intensive care physicians report death determination after cardiac arrest	Only 39% of surveyed physicians use various combination of clinical tests conforming to ANZICS definition of death. About two-third of respondents had heard about autoresuscitation and 37% had seen one
Dubois et al. USA 1999 [23]	To assess views of medical personnel regarding brain death and organ retrieval and related issues	Only 25% agreed to declare a person dead and retrieve organs prior to the death of brain if heart and lungs have stopped functioning for a few minutes. 62% supported the claim that higher brain death is death and 61 and 63% of participants supported organ retrieval from anencephalic patients and from higher brain death respectively
ElSafi et al. Saudi Arabia 2017 [26]	To explore the knowledge and attitudes toward organ donation and transplantation among 1st-year pre-clinical students before their taking any health science courses compared with students taking more advanced courses	Majority do not support deceased organ donation and 49.9% mentioned mistrust of the medical staff regarding brain death diagnosis as a reason
Floden et al. Sweden 2011 [9]	To present data on Swedish ICU nurses’ attitudes to brain death and organ donation and to test a questionnaire designed to explore these issues in terms of validity and reliability	48% of nurses trusted brain death diagnosis without confirmatory cerebral angiography, whether this reflects Knowledge gap or lack of trust it is unclear
Goudet France 2013 [44]	To determine the ethical acceptability for a large population of hospital personnel of organ donation following uncontrolled cardiac death	65% of the respondents thought that care givers might find it hard to reconcile the two aims of prolonging life vs organ preservation in the setting of uncontrolled cardiac death. 56% of these HCW find some aspect of uncontrolled DCDD problematic
Hart et al. USA 2012 [45]	To identify factors related to critical care physicians’ and nurses’ willingness to help manage potential donors after circulatory determination of death, and to elicit opinions on the presence of role conflict caring for donors after circulatory determination of death and its impact on end-of-life care	Minorities of physicians (14.7%; 95% CI 12.0–17.4) and nurses (14.3%; 95% CI 11.0–17.6) believed that managing DCDD would create professional role conflicts
Health professionals survey Canada 2006 [51]	To develop an understanding of Canadian healthcare professionals’ awareness, attitudes, and beliefs surrounding organ and tissue donation; To discover Canadian healthcare professionals’ views on donation after cardiocirculatory death including family/legal/ethical issues	Surveyed health care professionals found it unacceptable to perform medical procedures or administer medications to the patient before or immediately after circulatory death, with the sole intention to preserve organs for transplantation without prior consent
Honarmand et al. 2020 [59]	Attitudes of HCPs involved in OD and transplantation towards cardiac transplantation via DCDD	In the open-ended responses concerns were expressed about certainty of death and implications of restarting the heart after death declaration 22% of respondents had concerns about interruption of cerebral vasculature during the NRP and 2/3rd of the respondents felt ethical concerns were a significant barrier in implementation of NRP protocol
Hu China 2015 [55]	To assess the knowledge, attitudes, and willingness toward organ donation among health professionals in China	68.9% thought brain death was a reasonable criterion to judge death
Hyde et al. Australia 2011 [31]	Examined negative donation perceptions and explored any potential differences in these beliefs in a sample of people who self-identified as donors (want to donate upon death), non-donors (do not want to donate), and undecided (uncertain about donation preference)	. 14.7% of participants believed the true definition of brain death to be false
Iriarte Spain 2012 [32]	Show whether there is confusion amongst students about brain death and to investigate whether teaching in medical schools could influence knowledge about brain death held by students	67% of nursing students believed a brain dead patient was in coma and still alive. Percentage of medical students who believed brain dead patient is dead varied with the year of schooling with lowest being 38% in 5th year and highest being 72% in 3rd year of medical school
Joffe et al. Canada 2008 [46]	To determine if university students consider the donation after cardiac death donor as dead	Less than half of the respondents consider the patients in the DCDD scenarios dead (45%) or consider the physicians truthful in describing the patients as definitely dead (52%)
Joffe et al. Canada 2008 [41]	To determine whether paediatricians consider the donation-after-cardiac-death donor as dead	Given scenarios of patients being dead as per current DCDD guidelines, < / = 60% of physicians considered patients as dead. Only 3.8% allowed DCDD despite disagreeing or strongly disagreeing that the patient was definitely dead suggesting general support for Dead Donor Rule
Joffe et al. USA 2012 [22]	To determine whether board-certified neurologists in the United States agree with the standard definition of death and understand the criteria and the empirical state of the brain diagnosed by the tests used to confirm BD	Most neurologists do not understand or disagree that certain brain functions, including EEG activity (70%), evoked potential activity (56%), cerebral blood flow (52%)and hypothalamic neuroendocrine function (9%), often can remain in patients diagnosed dead using accepted tests. This suggests that these neurologists think that clinical tests for BD produce many false-positive diagnoses of death
Keenan et al. Canada 2002 [56]	To determine the attitudes toward organ donation from non–heart-beating cadaver donors in a sample of the general public and health care workers	Both the general public and health care workers support the use of non-heart-beating cadaver donors once a decision has been made to withdraw life support
Kubler et al. Poland 2009 [33]	To evaluate the attitude of university students to the concepts of brain death and organ retrieval, compared with the attitude of critical care physicians	98.6% of physicians know BD is legally dead, however 27.3% would consider brain dead as good as dead. 11.8% of physicians classified correctly severely brain injured person as alive but were willing to donate organs. The corresponding figure for patients in vegetative state was 8%
Lee et al. 2018 [57]	Relationship between attitudes to DCDD and palliative medication prescription among intensive care physicians	38% were concerned that DCD patients would receive inappropriate doses of palliative care medications Some thought prescribing high doses of palliative medications would be perceived as hastening death
Lewis et al. 2020 [60]	Attitudes of American Muslim HCPs to BD and its relationship with religiosity	84% of Muslim Allied Health Professionals believe that a person declared brain dead according to the American Academy of Neurology guidelines is truly dead
Lomero et al. Spain 2015 [24]	Attitude and knowledge regarding donation and transplantation of the medical and nursing staff at a community hospital in the province of Barcelona	69.1% agreed with the view that brain death is equivalent to death
Marck et al. Australia 2012 [30]	A cross-sectional survey was conducted to assess Australian ED clinicians’ acceptance and knowledge regarding BD	The majority (85.5%, n = 578) of respondents accepted BD, agreeing that “BD is a valid determination of death,” while 11% (n = 73) disagreed, and 4% (n = 25) replied “don’t know.” 37% of those who replied disagreed or don’t know said so due to “doubts on the scientific definition of BD”
Marcum USA 2002 [14]	The purpose of this study was to investigate operating room nurses' knowledge level of the organ transplant retrieval process and their attitudes toward organ donation	20% of operating room nurses disagreed with the definition of brain death
Mathur et al. USA 2008 [42]	Perception, level of knowledge, and understanding of DCDD and the effect of an educational intervention	Good support (82%) for giving medications such as heparin to improve the chances of successful donation after cardiac death. 37% were neutral and 14% disagreed that 5 min of unresponsiveness, apnoea and asystole are sufficient to pronounce death after withdrawing life support therapy
Mikla et al. Poland 2015 [11]	To analyze the knowledge and acceptance of the brain death (BD) concept among nursing students	(n = 369) knew the concept of BD and considered it to mean a person’s death. Of the rest, 19% (n = 93) did not know it, and the remaining 6% (n = 30) believed that it did not mean that a person was dead
Nair-Collins et al. USA 2015 [49]	To evaluate the public’s opinion about organ removal if explicitly described as causing the death of a donor in irreversible apneic coma	19–38% of people willing to donate their organs after death were either unsure or unwilling to donate their organs in the circumstance of irreversible apneic coma with organ removal causing biological death
Nasrollahzadeh et al. Iran 2003 [27]	To examine the critical items that influence nurse knowledge regarding the concept of brain death and attitudes toward cadaveric donor renal Tx	67% understood BD = death, but only 40% understood true concept and importance to BDD
Nowak et al. Poland 2014 [34]	Assessed young people’s knowledge and attitudes towards determining death in transplantology and their impact on attitude toward organ transplantation	85% of medical students and 54% of nonmedical students considered BD as the death of a human being, and the majority of the remaining group was reluctant to form a final opinion about this statement rather than simply deny it
Oo et al. 2020 [61]	Attitudes and knowledge of Malaysian ICU nurses regarding OD and BD, and relationship with sociodemographic attributes	12.1% of Malaysian HCW were not convinced or unsure of the clinical state called brain death
Othman et al. 2020 [38]	International study comparing public opinion to BD vs DCDD	87.9% of participants exposed to the circulatory death vignette were certain that the patient was truly dead vs 84.1% in the group exposed to the brain death case vignette a small but significant difference (Cohen's d 0.176; p = 0:004
Public survey Canada 2005 [37]	To survey the general public on awareness, attitudes and behaviours related to organ and tissue donation including the issue of donation after cardiac death	16% found the fact that surgery can start 5 min after heart stopping as unacceptable, 24 and 30% found the it unacceptable to perform procedures or administer medications to preserve organs respectively 20% believed doctors may prematurely declare a patient dead in order to get a potential organ donation
Rodrigue et al. USA 2018 [48]	To develop a questionnaire to measure health-care providers’ DCDD attitudes that can be quantified and standardized for use in research, quality improvement, and educational contexts	31% felt less comfortable with DCDD as compared to BD organ donation and 16% felt that the time of asystole or pulselessness to declare death in context of DCDD is too short. 11% felt death is declared too soon in DCD
Rodriguez-Arias Spain France USA 2013 [47]	Health professionals’ experience, beliefs and attitudes towards brain death and two types DCDD—controlled and uncontrolled	94% of HP believed patient who is BD is dead. This figure was 84% for uncontrolled DCDD and fell to 57% for a scenario of controlled DCDD. 55–60% of HP thought it was necessary to demonstrate BD in the DCDD scenarios
Roels et al. multiple countries 2010 [20]	Impact of Critical Care staffs’ attitudes to organ donation, their acceptance of the BD concept, their self-reported skills and educational needs on national donation rates	Support for the statement ‘Brain death is a valid determination of death’ was the highest in Norway (94.7%) and Belgium (89.7%) and the lowest in Croatia (67.4%) and Japan (36.4%) (average: 79.4 ± 16.3%). In each country and on average, acceptance of the BD concept was lower amongst nursing staff. Acceptance had a strong correlation with retrieval efficiency index
Rozaidi et al. Malaysia 2000 [28]	1. The concept of brain death 2. Withdrawal and the discontinuation of life support in brain dead patients 3. The acceptance of cadaveric organ donation and transplantation	83.8% accepted BD; 8.5% rejected it, 7.7% unsure. The reasons for not accepting were mostly religious beliefs and the perception of lack of evidence around the concept
Sarnaik et USA 2013 [39]	Views of pediatric intensive care physicians on the ethics of pediatric donation after cardiac death	25% of the participants believe DCDD donors feel pain during the harvest procedure as Anaesthesia is not administered
Schicktanz et al. Germany 2017 [35]	Attitudes towards organ donation, medical and economics students	More than 55% of individuals don’t agree or don’t know if the person is dead after brain stops functioning completely. 28.2% of people surveyed believed the person is dead if the regions of brain controlling personality thinking and speech are irreversibly destroyed
Siminoff et al. USA 2004 [36]	Public attitudes and beliefs about the determination of death and its relationship to organ transplantation	one-third (33.7%) believed that someone who was brain dead was legally dead, 43.3% referred to brain dead patients " as good as dead" while 16.3% considered them alive. 33.5% were willing to donate the organs of patients they classified as alive seemingly in violation of dead donor rule. 57.2% identified the patient in a coma as dead, and 34.1% identified the patient in a PVS as dead
Skwirczyńska et al. 2019 [58]	Attitudes to and knowledge of DCDD compared with BD among intensive care medical staff in Poland	79% of respondents declared acceptance of neurologic criteria as adequate to diagnose death in the case of a potential organ donor, 12% of respondents indicated circulation criteria, and only 9% declared both criteria as suitable for the diagnosis of death. A considerable percentage of respondents (79%) do not accept equivalent consideration of cardiovascular and neurologic criteria as suitable for diagnosing the death of a potential donor
Teixeira et al. Brazil 2012 [12]	Influence of understanding of brain death on organ donation	The majority of the population under study does not understand the meaning of BD and believes that the deceased potential donor might yet live. Trust in the diagnosis was directly correlated with favourable opinion towards organ donation. There was no statistical correlation between the level of education and the lack of understanding of BD
Yang et al. China 2015 [29]	To better understand the factors influencing the Chinese perception of brain death	34.1% found brain death ethically unacceptable. Only 50.7% considered a patient presented in a brain-dead scenario as dead, 51.9% were willing to withdraw
Youngner et al. USA 1989 [21]	Knowledge of, and concepts about, (1) the determination of death among physicians and nurses most likely to become involved in the identification and medical management of potential donors, (2) the discussion of the donation option with families, or (3) the actual retrieval of organs	95% considered loss of all brain function as death. 38% of respondents expressed irreversible loss of cortical function i.e. higher brain death concept as death. Many more with a higher brain concept as compared to lower/or whole brain concept thought that it was morally permissible to retrieve organs from patient who had lost all cortical function (68% vs 11%, *P* < .001)

### Attitudes to BD

32 studies examined attitudes to BD. The large majority surveyed healthcare professionals, with a second group involving university students. Only 6 studies surveyed the general public, one of which was mainly in the form of a satisfaction survey among donor families. 19 studies directly addressed whether acceptance of BD was broadly consistent with the death of a person. In most populations studied, around 75% accepted this proposition, though there was considerable variation. Several studies noted substantial numbers of respondents who supported a ‘higher brain’ concept of death, while others noted a willingness to proceed with OD even where respondents believed a hypothetical patient was still alive. Five studies noted either distrust of doctors or distrust of clinical techniques of BD determination.

#### Studies involving healthcare workers

One of the largest studies [20] was conducted in 245 hospitals across 11 countries, involving critical care staff reporting attitudes towards brain death and its correlation with organ donation. This revealed that support for the statement ‘Brain death is a valid determination of death’ was highest in Western Europe and lowest in Japan (Norway 94.7%, Belgium 89.7%, Croatia 67.4%, Japan 36.4%). Acceptance of the BD concept was significantly lower among nursing staff (77.4 ± 17.3%) compared with physicians (87.2 ± 9.75%). Average national medical and nursing staff acceptance of BD showed a strong positive correlation with national organ donation rates.

In North America, Youngner et al. [21], in a 1989 Cleveland study, interviewed 195 medical and nursing staff considered likely to be involved in organ retrieval. 58% did not use a coherent concept of death consistently. 19% had a concept consistent with a ‘higher brain’ definition of death. Joffe et al. [22] surveyed 218 US neurologists regarding their understanding of BD. 48% equated irreversible loss of consciousness with death. Many also believed that persistence of brain-mediated hormonal function was not compatible with a diagnosis of BD. In a study by DuBois et al. [23] 63% of participants agreed to organ retrieval from patients with ‘higher brain’ death.

In Europe, Floden et al. [9] surveyed 702 Swedish intensive care nurses. Less than half trusted the clinical diagnosis of brain death without additional imaging techniques. In a recent survey of 146 Spanish nurses, Lomero et al. [24] found that 69% equated BD with death.

In the Middle East, Alsaied et al. [10] surveyed 418 healthcare workers in Qatar. While a majority supported organ donation, less than half equated BD with death of the person. Cohen et al. [25] surveyed 2336 healthcare professionals involved in organ retrieval in Israel. 78.9% regarded BD as a valid criterion for determining death. Increasing age, higher professional status and working in ICU correlated with acceptance of BD. Acceptance correlated with greater comfort in the OD process. El Safi et al. [26] surveyed 434 allied health students in Saudi Arabia. Only 44% supported deceased OD, though 83% supported living OD. 49% did not trust medical staff regarding the diagnosis of BD. Nasrollahzadeh et al. [27] surveyed 130 Iranian ICU nurses. 67% accepted BD as death.

In Asia, in a Malaysian survey of medical and nursing staff [28], 83.8% accepted the concept of BD. Of those who did not, most cited either religious reasons or claimed there was insufficient scientific evidence to support the concept. In a 2015 Chinese study of 476 doctors and nurses [29], only 50.7% considered a hypothetical BD patient dead, 51.9% would withdraw support and only 40.6% would support organ retrieval.

In Australia, Marck et al. [30] surveyed 811 Australian emergency department clinicians. 86% accepted BD as death.

#### Studies involving university students

In a survey of 468 Australian university students and members of the general public by Hyde et al. [31], more than 30% of respondents unwilling or undecided about OD believed that BD patients had potential for recovery, while only 10% of willing donors agreed with this. Iriarte et al. [32] surveyed 536 Spanish university students. Less than 1/3 of non-medical students identified BD as death, and even among final year medical students, only 2/3 accepted BD as death.

Three Polish studies have addressed this question. Kubler et al. [33] surveyed 989 Polish university students. 48% believed a hypothetical BD patient was still alive, and half overall supported OD. In a study by Mikla et al. [11] of 492 Polish nursing students, 75% accepted BD as death. Nowak et al. [34] found that 85% of Polish medical students and 54% of non-medical students equated BD with death. Investigators also found high levels of mistrust of the diagnostic criteria for BD and for the skill and objectivity of doctors making the diagnosis.

In a 2017 German survey of medical and economics university students by Schicktanz et al. [35], around 44% agreed that when a person’s brain completely stops functioning, that person is dead.

#### Studies involving the general public

Siminoff et al. [36] conducted a telephone survey of 1351 Ohio residents in 2004. 86.2% regarded a hypothetical BD patient as dead, while 57.2% regarded a comatose patient as dead, and 34% regarded a vegetative patient as dead.

In Brazil, Teixeira et al. [12] found that 77% of hospital patients interviewed did not think of brain death as death, and there was no statistical correlation between respondents’ education and their understanding of brain death. As in Nowak’s study of Polish students [34], high levels of mistrust in the diagnosis of brain death was also found. 26.5% did not trust and 55.1% partially trusted the diagnosis of brain death. Likewise, in a 2005 study undertaken by the Canadian Council for Donation and Transplantation [37], 20% of respondents believed doctors might prematurely declare death in order to obtain organs for transplantation.

Othman et al. [38] studied 1072 people in 30 counties. In their study, respondents were more likely to accept circulatory death as death of the person than for brain death (87.9 ± 19.7% vs 84.1 ± 22.7%, *P* = 0.004). However, this was not reflected in a difference in acceptance of OD.

### Attitudes to DCDD

Fourteen studies examined attitudes to DCDD (Table 3). Once again, the large majority involved healthcare workers, with only 4 studies surveying the general public and one involving medical students.

The most common issue identified was concern with the duration of circulatory arrest required to determine death.

Sarnaik et al. [39] surveyed 273 American paediatric intensivists. 41% expressed concern that the timing of death during DCDD could not be precisely determined. Dhanani et al. [40] surveyed 250 Canadian intensivists. They reported variability in the determination of death after cardiac arrest, concerns regarding autoresuscitation, and a perceived need for standardisation of practice.

Joffe et al. [41] surveyed 80 paediatricians in a Canadian university children’s hospital. Almost half expressed concern that a hypothetical DCDD patient could not be regarded as unequivocally dead after 5 min of circulatory arrest. In a survey of 93 US paediatric critical care nurses by Mathur et al. [42], 14% believed that a 5-min observation period after circulatory arrest was insufficient to declare death.

De Jong et al. [43] interviewed 189 members of the general public in Canada, asking how long after circulatory arrest a hypothetical patient could be regarded as dead. After 5 min of arrest, 53% agreed that death had occurred and 42% agreed that the heart could be removed for transplantation. Where the heart had stopped ‘mere seconds ago’, 46% still agreed death had occurred, but only 24% agreed with removal of the heart.

Three studies reported more general concerns with the diagnosis of death in DCDD or the DCDD process. Goudet et al. [44] surveyed 1057 French healthcare professionals. 54% reported ethical concerns with DCDD, with junior intensive care doctors reporting the greatest level of concern. Hart et al. [45] carried out a US national survey of 684 intensivists and 438 ICU nurses. Around 14.5% of both groups expressed concern that the management of DCDD patients could create professional role conflicts, though 33.8% of physicians and 55.1% of nurses believed DCDD could potentially improve end-of-life care. Joffe and colleagues [46] surveyed 320 university students from a number of disciplines, finding that they too were not confident that a hypothetical DCDD patient was actually dead. Rodriguez-Arias et al. [47] interviewed 587 healthcare professionals involved in organ retrieval in Spain, France and the US. Main themes identified were that BD was regarded as a more reliable standard for the diagnosis of death in organ donors than circulatory death, and, while most regarded organ retrieval from brain dead patients as morally acceptable, DCDD was much more contentious.

In a study by Rodrigue et al. [48] in the US, 15% of the critical care staff were not sure if a patient is dead at the time of organ recovery in DCDD cases.

### Attitudes to the DDR

Six studies directly or indirectly raised issues concerning the DDR (Table 3). Only 3 involved the general public, all North American.

In the Polish student study by Kubler [33] 34% of respondents supported OD from non-brain-dead unconscious hypothetical patients. In the German study by Schicktanz et al. [35], 28% of students supported a’higher brain’ definition of death. In Siminoff’s Ohio study [36], 33.5% of the general public supported OD in cases they did not regard as dead.

In De Jong et al.’s survey of the Canadian public [43], 49% of respondents agreed that the DDR should be abandoned and 58% agreed that different definitions of death should be used for organ donation. However, in Sarnaik’s [39] study of US intensivists, 84% supported the principle of the DDR.

Nair-Collins[49] surveyed 1096 members of the American general public in 2015, using a scenario involving an irreversibly comatose patient, where it was explicitly stated that organ donation would cause death. 71% of respondents agreed it should be legal for patients to donate organs in this situation. Of those generally willing to donate their organs, 76% agreed they would donate in these circumstances.

### Ante-mortem interventions and consent

We identified 6 studies that examined attitudes to ante-mortem interventions in DCDD. Only one involved members of the general public.

Camut et al. [50] surveyed 173 French healthcare professionals in 2013 regarding the provision of non-therapeutic intensive care in a case of massive stroke, for the purpose of organ donation. 93% of respondents believed this was acceptable, but 75% required advance consent of the patient and their family. The findings of a Canadian survey of health care professionals [51] echoed similar views—a majority of them found it unacceptable to perform medical procedures or administer medications to the patient before or immediately after circulatory death, with the sole intention to preserve organs for transplantation without prior consent.

In the Canadian Council for Donation and Transplantation study [37] only half the respondents found medications and procedures provided before death to maintain organs acceptable.

Support for interventions seemed to vary depending on the degree of invasiveness. In the study by Dhanani et al. [40], while there was overwhelming support for heparin infusion to preserve organs, that support diminished when cannulation was considered.

In a study by Goudet et al. [44] 42% of respondents did not want cannulation of the patient for organ preservation without prior family consent. An important significant minority regarded this as an unacceptable alteration of body integrity. Similar findings were noted in the study by Sarnaik et al. [39].

## Discussion

This scoping review examined evidence regarding acceptance of and attitudes towards the concepts of BD, DCDD and the DDR, and how these relate to attitudes and decision-making regarding organ donation. We found that there is strong support for OD, but a range of views regarding BD, DCDD and the DDR—both within and between different countries and populations—with persisting concerns regarding the extent to which BD represented death of the person. In one study, organ donation rates of a country correlated positively with acceptance of BD [20]. A substantial proportion of respondents in several studies appeared to favour a ‘higher brain’ concept of death, while others were comfortable with OD, even if it was the proximate cause of death [35, 36].

A striking feature of our review was the paucity of studies examining attitudes of the public, with a large majority involving healthcare workers of various types. A second, smaller group of studies focussed on university students, mainly comprising medical and nursing students. This lack of more broadly-based information is important, because it may help to explain disparity between the high reported rates of support for OD and the relatively low rates of consent reported in many jurisdictions.

Another notable feature was the tendency to ascribe rejection of or uncertainty about these concepts of death to a knowledge deficit that could or should be addressed by further education—a well-recognised assumption in health care and public policy debates known as the ‘knowledge deficit model’ of the public understanding of science. This is a problematic assumption both because it fails to recognise that differences of opinion may represent genuine differences in values and because there is considerable data suggesting that while knowledge and education may predict the *strength* of attitudes to scientific matters, positivity of attitudes are poorly correlated with knowledge [52].

While the diagnosis of BD has been widely accepted medically and legally as equivalent to death of the person for over 50 years, our review revealed that 20–40% of participants in most studies do not accept that BD is truly equivalent to death of the person. Some studies showed that age, education and background in healthcare were associated with a higher likelihood of accepting BD as equivalent to death, but these features were not predictive. Religious or cultural factors on opinion could be implicated in some studies, but not in all.

Some studies [36] found sizeable proportions of respondents who considered that severe brain injury not meeting the accepted criteria for BD was sufficient to determine death. Even among American neurologists, when asked to give a reason why brain death is equivalent to death, 48% chose a ‘higher brain’ explanation [22]. This is an important finding, as such levels of brain injury are not accepted as the basis for determining death in any jurisdiction.

In comparison to studies examining attitudes to organ donation after BD (DBD), we found far fewer studies examining attitudes to DCDD. Importantly, most of these studies found less support for DCDD than DBD. The principal issue of concern appears to be the timing of determination of death, with around half the respondents to most surveys expressing discomfort with the idea that a few minutes of cardiorespiratory arrest were adequate to determine the death of an individual. Once again, however, these studies were predominantly conducted in healthcare workers rather than members of the general public.

Lack of confidence in medical procedures or in medical practitioners around the diagnosis of death were frequently noted in relation to both BD and DCDD. In studies that examined confidence in the methods used to diagnose BD, a substantial number of respondents did not have full confidence in either the doctors making the diagnosis, or in the diagnostic criteria or tests used. In the case of DCDD, the most common issue identified was lack of confidence regarding whether the very short time after which death was being determined following cessation of circulation, could be considered accurate. The potential for a conflict of interest between the desire to procure organs for transplantation and the requirement to provide appropriate palliative care to a patient at end of life was also noted as a concern in DCDD cases.

Only a few studies explicitly reported attitudes to the DDR, and in all, considerable proportions of respondents supported retrieval of organs for transplantation from patients with severe brain injury who were not BD. In these studies, the proposition put to respondents was that brain death was not determined prior to organ donation, or donation was not occurring following cardiorespiratory standstill as in DCDD. This is an important, and for some possibly an uncomfortable finding, as it suggests that for many people, life with severe brain dysfunction and poor prospects for a sentient and relatively independent future would be considered as being of less ‘value’ than donating organs and thus dying. In this setting the physiological and clinical criteria by which death is determined in medical practice would appear to have little relevance. Of interest, the study with the lowest level of acceptance of organ donation in patients without BD was the only study focussed entirely on medical professionals, suggesting perhaps that the principal discomfort with these concepts lies within the healthcare community.

Antemortem interventions were only considered in 6 studies, 5 limited to healthcare professionals, and all found levels of discomfort, with most respondents insisting this was only acceptable with explicit consent, especially for invasive procedures. In this context, it is interesting to note the study by Shahrestani et al. [53], who interviewed 30 clinicians involved in transplantation from 8 countries. From their thematic analysis, they concluded that ante-mortem interventions were acceptable only where distress for the donor and family are not increased, the interventions did not cause harm, patient and family have a strong drive to successful donation, and the interventions are evidence-based.

### Strengths and limitations

We conducted a comprehensive search to review all English language, quantitative studies involving attitudes and beliefs surrounding BD and DCDD in the context of organ donation. A scoping review allows a broader range of studies to be included than a systematic review. While it does not provide the same statistical rigor, it is preferable where it is not appropriate to aggregate divergent datasets for meta-analysis. Our review was guided by the PRISMA protocol to ensure our sample captured all the relevant scholarship. Our review consolidates a vast international literature on attitudes toward BD and DCDD in the context of organ donation. It brings to light the divergent attitudes about how death is determined before organ donation, despite strong support for organ transplantation generally.

These results are limited by a few factors. A significant limitation was the tendency for studies to conflate attitudes with knowledge. We suggest that attitudes relate more to socio-cultural values than factual knowledge, though we identified no data to support this. Non-English literature was excluded from our review but could communicate different perspectives than the ones reported herein. Healthcare workers directly involved in organ donation, whose attitudes are more relevant to clinical practice, have been poorly studied. We propose to address this in future studies. Finally, notwithstanding our assessment of bias, the findings of the studies in this review could still be subject to biases inherent in all questionnaire based studies [54]

## Conclusion

The idea that death is a prerequisite to the removal of vital organs for transplantation has been an ethical cornerstone of medical practice since transplantation began. However, there is a fundamental tension between the need to minimise ischaemic time to ensure successful transplantation, and the need for death to be confidently diagnosed before transplantation can proceed. This tension has largely driven changes in the way death is diagnosed in this context, resulting in the widespread adoption of the concepts of BD and, more recently, circulatory death and DCDD. These innovations have been promulgated by those directly involved in transplantation and organ donation, with little effort to assess their acceptance among the health professions, or the general community.

Our review suggests that a considerable proportion of healthcare workers, as well as members of the general public, have doubts about the conceptual and clinical validity of BD and DCDD as ways to determine death, especially before organ donation. These doubts are usually ascribed to ignorance about BD and/or DCDD, or to ‘unjustifiable’ or ideological opposition to them. However, the fact that these concerns are expressed across different populations and cultural contexts and are voiced even by experts in the field, including intensive care professionals, suggests that these explanations may be unfounded.

Likewise, a considerable proportion of people appear to feel prognosis (meaning the likelihood of a return to meaningful or quality life following brain injury), rather than the diagnosis of death per se, is most important regarding decisions about organ donation and the cessation of ICU support. This suggests the need to (re)engage the public in discussions about the values and goals of medical care and move away from the idea that debates about end-of-life care can be simply resolved by clarifying and promulgating different definitions of death.

Finally, studies examining attitudes to perimortem interventions suggest that these are only acceptable following explicit consent, and where the consequences for the donor are minimal.

Further studies are needed to examine the complex interplay of factual knowledge and values-based attitudes regarding death in determining the overall acceptance of organ donation.

### Supplementary Information

**Additional file 1**. Literature Search Strategy.

## Data Availability

Not applicable.

## Appendix

**Table:**

Brain death (BD)	A definition of death as complete and irreversible loss of brain function, even when the circulation and breathing are maintained by external means
Defibrillation	The use of electrical stimulation to restore heart contractions when they have ceased
Cardiac/circulatory/respiratory death	A definition of death as the complete cessation of heart and respiratory activity beyond a defined time interval
Dead donor rule (DDR)	An ethical principle stipulating that vital organs should only be removed for transplantation after a patient has been declared dead
Ischaemic injury	Damage to organs and tissues that develops progressively when they are deprived of blood flow
Auto-resuscitation	The spontaneous re-commencement of cardiac and/or respiratory activity some time after these have ceased
‘Higher brain’ concept of death	A definition of brain death as the irreversible loss of the capacity for consciousness
Antemortem interventions	Medical interventions administered to a prospective organ donor prior to death, in order to prepare or preserve organs for transplantation
