# Supplementary material for: A scoping review of the perceptions of death in the context of organ donation and transplantation
Source: BMC Med Ethics. 2021 Dec 18;22:167. doi: 10.1186/s12910-021-00734-z (PMC8684159; doi:10.1186/s12910-021-00734-z)
Supplement: Supplementary file 1 — Additional file 1. Literature Search Strategy. [file 12910_2021_734_MOESM1_ESM.docx]

**A scoping review of the changing perceptions of death in the context of organ donation and transplantation**

**APPENDIX – SEARCH STRATEGY**

**Table 1. Medline search strategy**

| **Title:** Changing perceptions of death in the age of organ donation and transplantation: a systematic review | | |
| --- | --- | --- |
| Sets 1-9 are the**MeSH terms &  text words [words found in the  TITLE or ABSTRACT of a record]**  They are combined using **OR** | 1. Brain Death [MeSH] 2. brain dead.mp 3. brain death.mp 4. heart dead.mp 5. heart death.mp 6. cardiac dead.mp 7. cardiac death.mp 8. circulatory dead.mp 9. circulatory death.mp | **Death** |
| Sets 11-16 are the **MeSH terms & text** **words [words found in the TITLE or** **ABSTRACT of a record]** for the  **intervention** (i.e helmets). They are  combined using **OR** | 1. Tissue and Organ Procurement [MeSH] 2. organ procur*.mp 3. organ donor*.mp 4. organ donat*.mp 5. organ reserv*.mp 6. organ preserv*.mp | **Organ donation** |
| Sets 18-35 are the**MeSH terms &  text words [words found in the  TITLE or ABSTRACT of a record]** | 18. Ethics, Medical [MeSH]  19.     Informed Consent [MeSH]  20.     Decision Making [MeSH]  21.     PERCEPTION[MeSH]  22.     jurisprudence [MeSH]  23.     liability, lega [MeSH]  24.    Clinical Protocols [MeSH]  25.     Ethics [MeSH]  26.     Religion [MeSH]  27.     Attitude of Health Personnel [MeSH]  28.     HEALTH KNOWLEDGE, ATTITUDES, PRACTICE [MeSH]  29.     Comprehension [MeSH]  30.     Culture [MeSH]  31.     PSYCHOLOGY [MeSH]  32.     ATTITUDE [MeSH]  33.     Surveys and Questionnaires [MeSH]  34.     survey* or questionnaire*.mp  35.     belief* or believe* or willing*.mp | **Perception** |
| Sets 37-47 are the**MeSH terms &  text words [words found in the  TITLE or ABSTRACT of a record]** | 37.    Physicians [MeSH]  38.    Health Personnel [MeSH]  39.     Healthcare provider*.mp  40.     Students, Medical [MeSH]  41.     Medical Staff, Hospital [MeSH]  42.     medical adj personnel*.mp  43.     Nursing Staff [MeSH]  44.     critical care nurs*.mp  45.     intensive care nurs*.mp  46.  ICU nurs*.mp  47. Public Opinion [MeSH] | **Perception** |
| **Each set was combined with & 10, 17, 36 and 48.**  **Search conducted on:**  **Database:** MEDLINE(R) and Epub Ahead of Print, In-Process & Other Non-Indexed Citations, Daily and Versions(R) 1946 to June 30, 2018   \| **Platform:** OVID \| \| --- \|   **Date Search conducted**: 30 June, 2018  **Search Conducted by**: Dianne Walton-Sonda, Senior Reference Librarian, ACT Health Library. | | |

**Table 2. Search vocabulary**

| EmTree terms (Embase/EmCare) | MeSH (Medline (OVID)/Cochrane/PubMed) | CINAHL Headings (CINAHL -EBSCO) | Thesaurus of Psychological Index Terms (PsycINFO OVID) |
| --- | --- | --- | --- |
| brain death | brain death | Brain Death | Brain death |
| heart death | Tissue and Organ Procurement | Organ Transplantation | Death and dying |
| organ transplantation | Ethics, Medical | Transplant Donors | Physicians |
| organ donor | Informed consent | Organ procurement | Health personnel |
| medical ethics | Decision making | Ethics | Health personnel attitudes |
| informed consent | Perception | Ethics, Medical | Professional ethics |
| decision making | Jurisprudence | Ethics, Nursing | Informed consent |
| perception | Liability, legal | Consent | Decision making |
| jurisprudence | Clinical protocols | Decision making | Perception |
| legal liability | Ethics | Jurisprudence | Professional liability |
| clinical protocol | Religion | Liability, Legal | Laws |
| ethics | Attitude to health personnel | Religion and Religions | Ethics |
| health personnel attitude | Health knowledge, attitudes, practice | Religion and Psychology | Attitudes |
| attitude to health | Comprehension | Attitude to health personnel | Health personnel attitudes |
| comprehension | Culture | Culture | Comprehension |
| cultural anthropology | Psychology | Psychology | Culture anthropological |
| psychology | Attitude | Attitude | Psychology |
| Religion | Surveys and questionnaires | Surveys | Religious beliefs |
| Attitude | Physicians | Questionnaires | surveys |
| Questionnaire | Health personnel | Physicians | Questionnaires |
| Health survey | Students, medical | Health Personnel | Physicians |
| Physician | Medical staff | Medical staff, hospital | Health personnel |
| Health care personnel | Medical staff, hospital | Medical staff | Medical students |
| Medical student | Nursing staff | Students, Medical | Nurses |
| Medical staff | Nursing staff, hospital | Nursing staff, Hospital | Social workers |
| Nursing staff | Public opinion | Public Opinion | Public opinion |
| Nursing |  |  |  |
| Intensive care nursing |  |  |  |
| Public opinion |  |  |  |
